# Modified geriatric nutritional risk index as a prognostic indicator for patients with intrahepatic cholangiocarcinoma undergoing liver resection

**DOI:** 10.3389/fnut.2026.1965547

**Published:** 2026-10-29

**Authors:** Moriyasu Takada, Thanh Tung Lai, Kyoko Inoue, Van Khanh Nguyen, Kosuke Matsui, Hidekazu Yamamoto, Hisashi Kosaka, Gozo Kiguchi, Hideyuki Matsushima, Takuya Ohigashi, Hoang Hai Duong, Kengo Yoshii, Masaki Kaibori

**Affiliations:** 1 Department of Hepatobiliary Surgery, Kansai Medical University, Osaka, Japan; 2 Department of Health and Nutrition, Faculty of Health Sciences, Osaka Aoyama University, Minoh, Japan; 3 Department of Surgery, Hanoi Medical University, Hanoi, Vietnam; 4 Department of Nutrition, Osaka Seiki College, Osaka, Japan; 5 Internal Gastroenterology Department, VNU University of Medicine and Pharmacy, Hanoi, Vietnam; 6 Department of Hepatobiliary and Gastrointestinal Surgery, Thai Nguyen National Hospital, Thai Nguyen, Vietnam; 7 Department of Mathematics and Statistics in Medical Sciences, Kyoto Prefectural University of Medicine, Kyoto, Japan

**Keywords:** hepatectomy, intrahepatic cholangiocarcinoma, modified geriatric nutritional risk index (mGNRI), prognostic factors, survival outcome

## Abstract

**Background:**

The modified Geriatric Nutritional Risk Index (mGNRI), which incorporates C-reactive protein (CRP) level and ideal body weight ratio, has been proposed as a prognostic indicator for survival in certain cancers. This study evaluated its association with outcomes in patients with intrahepatic cholangiocarcinoma (ICC) undergoing hepatectomy.

**Methods:**

We retrospectively analyzed 99 patients with ICC who underwent hepatectomy between April 2008 and August 2022. The optimal mGNRI cut-off (88.2) determined by receiver-operating characteristic (ROC) curve analysis, classified patients into low and high mGNRI groups.

**Results:**

Forty-five patients (45.5%) were in the low mGNRI group, and 54 (54.5%) were in the high mGNRI group. The low mGNRI group had a significantly lower body mass index (BMI) and hemoglobin and serum albumin levels, but higher CRP levels compared with the high mGNRI group. Recurrence-free survival (RFS; hazard ratio [HR]: 2.09, 95% confidence interval [CI]: 1.29–3.39, *p* = 0.003) and overall survival (OS; HR: 2.25, 95% CI: 1.32–3.83, *p* = 0.003) were both worse in the low mGNRI group than in the high mGNRI group. Multivariate Cox proportional hazards analysis showed that a low mGNRI was an independent prognostic factor for OS (HR: 1.86, 95% CI: 1.03–3.37, *p* = 0.041). Postoperative dietary intake was significantly lower in the low vs. the high mGNRI group.

**Conclusions:**

The mGNRI has a prognostic impact on patients with ICC after hepatectomy. Patients with a low mGNRI showed poorer survival and a lower postoperative oral energy adequacy ratio, highlighting the importance of perioperative nutritional assessment in this population.

## 1 Introduction

Intrahepatic cholangiocarcinoma (ICC) is the second most common type of primary liver cancer, accounting for approximately 10–15% of cases (1). The incidence of ICC varies by geographic region, race, and gender, but it is increasing worldwide (2–5). Since ICC has almost no initial symptoms, it is difficult to detect early, and it is often diagnosed when it has progressed. Furthermore, ICC is highly invasive, and surgical resection is the only curative treatment. However, ICC is a malignant disease with a poor prognosis, with approximately 60% of patients who have undergone curative treatment experiencing recurrence or metastasis (6, 7).

In cases of surgical resection for ICC, it has been reported that in patients with malnutrition before surgery, rates of postoperative complications and mortality are high, and this affects the long-term prognosis (8, 9). It has also been reported that preoperative improvement of malnutrition in patients undergoing hepatic resection can promote wound healing, improve immune function, reduce weight loss, and decrease rates of postoperative complications and mortality (10). Therefore, it is important to assess nutritional status correctly and manage nutrition during the perioperative period. Moreover, ICC is frequently accompanied by systemic inflammation and cancer-associated metabolic disturbances, which can further exacerbate malnutrition and impair postoperative recovery. Thus, nutritional assessment tools that also reflect inflammatory burden may provide superior prognostic information in this population.

The Geriatric Nutritional Risk Index (GNRI) is a nutritional assessment tool calculated from serum albumin levels and the ratio of actual to ideal body weight and is widely used to assess nutritional status and predict prognosis. Recently, a Modified Geriatric Nutritional Risk Index (mGNRI) that incorporates C-reactive protein (CRP) instead of serum albumin was developed. The mGNRI has been reported to be a better prognostic factor for esophageal and pancreatic cancer than the GNRI (11, 12). Unlike serum albumin, which is strongly influenced by inflammation and hepatic dysfunction, CRP is a sensitive marker of systemic inflammation and may more accurately reflect cancer-related catabolic states. Therefore, the mGNRI is considered a more comprehensive indicator that captures both nutritional status and inflammatory activity, which are key determinants of outcomes in patients with malignancy. However, no reports exist on the relationship between the mGNRI and prognosis in patients with ICC. In this study, we aimed to investigate the association between the preoperative mGNRI and long-term outcomes in patients with ICC who underwent liver resection. Furthermore, no study has investigated whether the preoperative mGNRI is associated with postoperative oral intake, an important determinant of recovery and long-term outcomes following hepatectomy.

## 2 Materials and methods

### 2.1 Patient selection

From April 2008 to August 2022, a total of 117 cases were histologically confirmed as ICC after liver resection at Kansai Medical University Hospital. Among these, 17 patients with mixed hepatocellular carcinoma and ICC and one patient with missing data were excluded. Finally, 99 eligible patients with ICC were retrospectively analyzed.

Surgical resection was indicated for patients with resectable ICC without extrahepatic metastasis, with preserved liver function and sufficient future liver remnant volume, based on multidisciplinary team evaluation. All surgeries were performed by the same hepatobiliary surgical team composed of board-certified and experienced hepatobiliary surgeons.

This study followed the Guidelines for Clinical Research provided by the Japanese Ministry of Health and Welfare. Approval of the study protocol was obtained from the Ethics Committee of Kansai Medical University (approval number: KMU 2019322). All procedures were performed in accordance with the principles of the Declaration of Helsinki.

The date of surgery for ICC was defined as the beginning of follow-up in this study. The follow-up period ended with the last recorded visit for surviving patients or the date of death during the observation period.

### 2.2 Follow-up

Postoperative surveillance was performed through outpatient visits, with follow-up evaluations including imaging studies and blood tests such as tumor marker assessments. Follow-up intervals were generally every 3 to 6 months during the first 2 years and every 6 to 12 months thereafter, in accordance with institutional practice. Clinical and pathological data were collected retrospectively from medical records. Tumor recurrence was diagnosed based on radiological findings on imaging studies, with supporting information from tumor marker levels and clinical course. Overall survival (OS) and recurrence-free survival (RFS) were defined as the length of time from the date of surgery to death and to the first documented recurrence, respectively, or to the date of last follow-up. Treatment after recurrence was determined according to institutional practice, considering tumor status, liver function, and patient condition.

### 2.3 Calculation of indices for estimating cancer prognosis

The mGNRI was calculated using the following formula: [14.89 / CRP (mg/dL)] + [41.7 × current body weight / ideal body weight] (12). The ideal body weight was calculated as 22 × height (m^2^). In cases where actual body weight exceeded the ideal body weight, this ratio was set to 1 (12). Because CRP level ≤ 0.3 mg/dL were reported as within normal limit, serum CRP ≤ 0.3 mg/dL was considered as 0.3 mg/dL in this study. The coefficient of 14.89 gives greater weight to the CRP component, resulting in a more balanced contribution of CRP relative to the body weight component in the overall mGNRI. The GNRI was calculated as 14.89 × albumin (g/dL) + 41.7 × current body weight / ideal body weight. The ratio of actual weight to ideal weight was set to 1 when the patient's actual weight exceeded the ideal weight (13). The CRP-to-albumin ratio (CAR) was calculated as CRP (mg/dL) / albumin (g/dL) (14). The CRP-albumin-lymphocyte (CALLY) index was calculated as [albumin (g/dL) × lymphocyte count (/μL)] / (CRP (mg/dL) × 10^4^) (15). The prognostic nutritional index (PNI) was calculated as 10 × albumin (g/dL) + 0.005 × lymphocyte count (/μL) (16). The platelet-to-lymphocyte ratio (PLR) was calculated as platelet count (/μL) / lymphocyte count (/μL) (17). The neutrophil-to-lymphocyte ratio (NLR) was calculated as neutrophil count (/μL) / lymphocyte count (/μL) (18).

### 2.4 Clinicopathological variables

The factors investigated in this study included patient characteristics, laboratory parameters, surgical factors, tumor-related factors, and dietary intake.

Patient characteristics included age, gender, body mass index (BMI), psoas muscle index (PMI), and presence of chronic viral hepatitis B or C. Laboratory parameters assessed included white blood cell (WBC), neutrophil, lymphocyte, and platelet counts, albumin, total bilirubin, aspartate aminotransferase (AST), alanine aminotransferase (ALT), alkaline phosphatase (ALP), CRP, carcinoembryonic antigen (CEA), and carbohydrate antigen 19-9 (CA19-9) levels, and the indocyanine green retention rate at 15 min post-injection (ICGR15). Preoperative blood samples and body weight measurements were routinely collected within 1 week prior to surgery during the final preoperative outpatient visit or upon admission. ICGR15, CEA, and CA19-9 were measured within 1 month prior to surgery.

Surgical factors examined included surgical approach (open or laparoscopic), type of hepatectomy classified according to the extent of liver resection (partial hepatectomy or segmentectomy and sectionectomy was classified as minor hepatectomy; whereas bisectionectomy or trisectionectomy was classified as major hepatectomy), extrahepatic bile duct resection, lymph node dissection, operative time, intraoperative blood loss, need for blood transfusion, tumor differentiation, tumor number, maximum tumor size, lymph node metastasis, serosal invasion, vascular and/or bile duct invasion, postoperative complications, American Joint Committee on Cancer (AJCC) classification, and use of postoperative adjuvant chemotherapy.

Lastly, factors related to dietary intake were assessed, including energy, protein, fat, and carbohydrate intake, as well as energy and protein adequacy ratios. Postoperative oral intake was evaluated as the average from postoperative day (POD) 3–6 to minimize perioperative variability.

### 2.5 Postoperative nutritional intake assessment

The amount of food intake per day was confirmed in the electronic medical record, and the intake of nutrients (energy, protein, fat, and carbohydrates) was calculated. Daily intake data were obtained from standardized nursing dietary records and verified by registered dietitians to improve accuracy.

Target nutritional intake (kcal/day) was calculated as follows: Target nutritional intake (kcal/day) = Basal Energy Expenditure (BEE) × Activity Factor (AF) × Stress Factor (SF). Two different formulas were used for BEE depending on sex. For men: BEE = 66.473 + 13.7516 × weight (kg) + 5.0033 × height (cm) −6.755 × age (years). For women: BEE = 655.0955 + 9.5634 × weight (kg) + 1.8496 × height (cm) −4.6756 × age (years) (19). The AF and SF were set from 1.0–1.3 and 1.0–1.2, respectively, by registered dietitians and physicians. The criteria for determining AF and SF are presented in Supplementary Table S1. The nutrient adequacy ratio (%) for each nutrient, including energy and protein, was calculated as follows: (nutrient intake / target nutrient intake) × 100.

### 2.6 Statistical analysis

Based on 3-year OS status, the prognostic ability of the variables was compared using the area under the receiver operating characteristic (ROC) curve (AUC) for the indicators that estimate nutritional status, immune status, and inflammatory status, with Youden's index applied. A time-dependent ROC analysis at 3-year OS was performed to evaluate the prognostic performance of mGNRI while appropriately accounting for censored observations. Internal validation was performed using 1,000 bootstrap resamples. In each resample, the optimal mGNRI cutoff was re-estimated using the Youden index from the 3-year time-dependent ROC analysis, followed by Cox regression for OS. The median and 95% bootstrap percentile intervals of the cutoff and HR were calculated. The other continuous variables were classified into two groups using median values.

The comparison of the characteristics of the high mGNRI and low mGNRI groups was conducted using Pearson's chi-square test or Fisher's exact test as appropriate. Differences between groups in energy adequacy ratio were assessed by a repeated-measures two-way ANOVA with Greenhouse-Geisser correction. Probabilities of RFS and OS after liver resection were calculated using the Kaplan–Meier method. Death from any cause was considered an event for RFS analysis. The hazard ratios (HR) and 95% confidence intervals (CIs) for RFS and OS were calculated using univariate and multivariate Cox proportional hazards analysis. The number of covariates included in each multivariable Cox model was determined based on the number of observed events. The covariates included Cox model was selected based their clinical relevance liver function, surgical, oncological factors. The proportional hazards assumption for the Cox models was assessed using Schoenfeld residuals, including tests for individual covariates and the global model.

For all analyses, *p*-values less than 0.05 were considered statistically significant. The statistical analysis was performed using IBM SPSS Statistics version 26 (IBM Corp., Armonk, NY, USA) for ROC curve analysis, which was used to evaluate the discriminatory ability of mGNRI and to determine the optimal cutoff value, and for repeated-measures two-way ANOVA analysis of variance. The other statistical analyses, including the time-dependent ROC analysis, were performed using R version 4.3.3 (R Foundation for Statistical Computing, Vienna, Austria).

## 3 Results

### 3.1 Comparison of the prognostic ability of indicators that estimate nutritional status, immune status, and inflammatory status with patient characteristics

Prognostic indicators were compared using ROC analysis based on 3-year OS status for indicators that estimate nutritional status, immune status, and inflammatory status (mGNRI, GNRI, CAR, CALLY index, PNI, PLR, NLR) (Figure 1). As a result, it was shown that mGNRI had the numerically highest AUC for predicting OS with a cutoff value of 88.2 (AUC = 0.648, 95% CI: 0.539–0.756, *p* = 0.012). Based on this exploratory cutoff value, 99 patients with ICC were classified into a high-mGNRI group (mGNRI ≥ 88.2, *n* = 54) and a low-mGNRI group (mGNRI < 88.2, *n* = 45). The characteristics of the two groups are shown in Table 1. The 3-years time-dependent ROC analysis for OS showed an AUC of 0.647, with a corresponding mGNRI cutoff value of 91.05.

**Figure 1:**
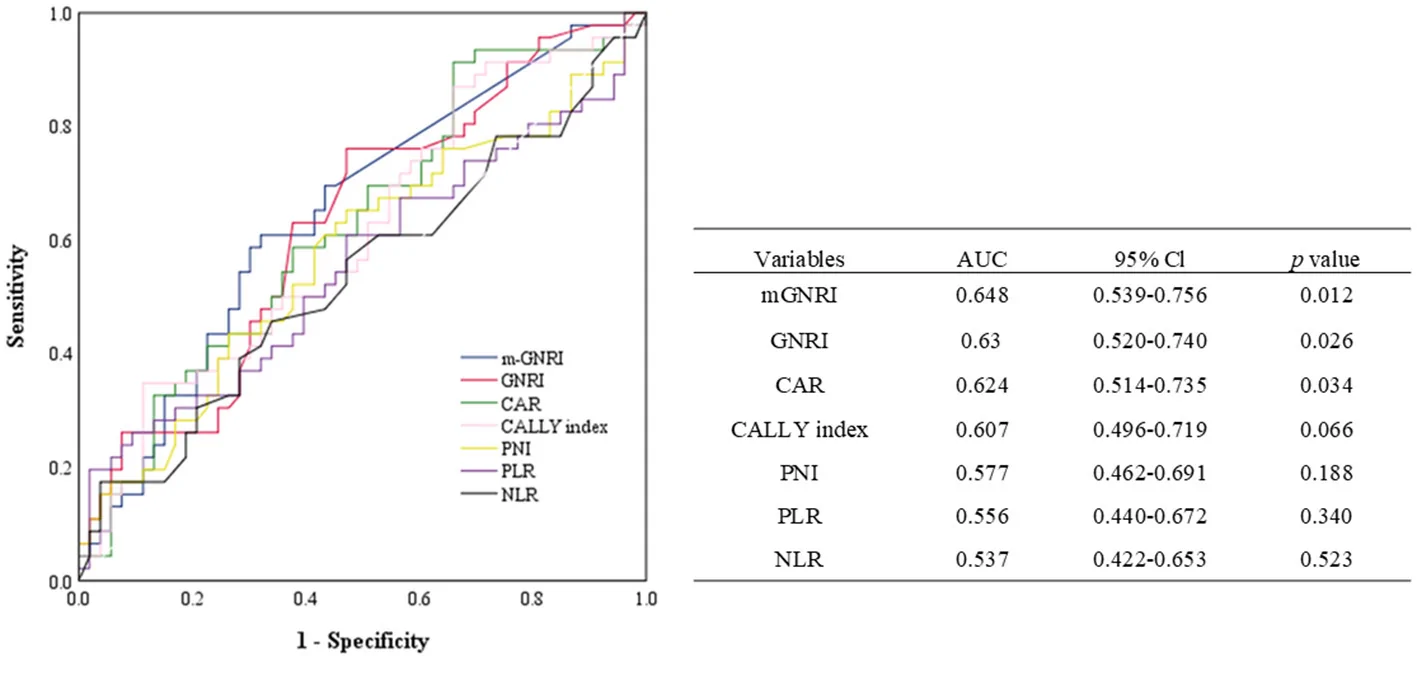
Receiver operating characteristic (ROC) curves for predicting overall survival based on nutritional and inflammatory indicators. The modified Geriatric Nutritional Risk Index (mGNRI) showed the highest area under the curve (AUC = 0.648, *p* = 0.012), showing the numerically highest AUC among the evaluated indices (GNRI, CAR, CALLY index, PNI, PLR, and NLR).

**Table 1:** Preoperative clinical and laboratory characteristics of patients with intrahepatic cholangiocarcinoma (*N* = 99).

Variable	High mGNRI (*n* = 54)	Low mGNRI (*n* = 45)	*p*-value
Age, years	72 (68–78)	72 (64–77)	0.552
Gender, male	36 (67%)	23 (51%)	0.172
BMI, kg/m^2^	24.6 (22.2–26.8)	21.0 (18.8–23.5)	< 0.001
PMI, cm^2^/m^2^	4.3 (3.6–5.5)	4.0 (3.5–4.7)	0.106
HBV, Positive	18 (33%)	10 (22%)	0.318
HCV, Positive	6 (11%)	2 (4%)	0.286
WBC, cells/μL	5,250 (4,675–6,500)	6,500 (4,950–7,600)	0.028
Neutrophil count, cells/μL	3,203 (2,475–4,367)	4,225 (3,162–5,247)	0.006
Lymphocyte count	1,391 (1,118–1,728)	1,274 (1,020–1,810)	0.586
Hemoglobin, g/dL	13.1 (11.7–13.6)	11.7 (10.9–12.6)	0.002
Platelet count, × 10^4^/μL	18.2 (13.6–23.0)	21.8 (16.2–26.9)	0.021
Albumin, g/dL	4.1 (3.8–4.4)	3.8 (3.2–4.1)	< 0.001
Total bilirubin, mg/dL	0.7 (0.6–0.9)	0.6 (0.4–0.8)	0.039
AST, IU/L	24 (20–30)	26 (20–32)	0.531
ALT, IU/L	19 (15–27)	18 (12–25)	0.243
ALP, IU/L	212 (148–276)	329 (217–510)	< 0.001
CRP, mg/dL	0.116 (0.049–0.196)	0.545 (0.163–1.560)	< 0.001
ICGR15, %	10.4 (7.9–14.3)	9.6 (7.6–16.1)	0.661
CEA, ng/mL	2.5 (1.6–4.4)	2.9 (1.7–5.7)	0.228
CA19-9, U/mL	29.0 (9.3–117.5)	38.2 (19.1–400.9)	0.049

Data are presented as median (interquartile range) or *n* (%). mGNRI, modified geriatric nutritional risk index; BMI, body mass index; PMI, psoas muscle mass index; HBV, hepatitis B virus; HCV, hepatitis C virus; WBC, white blood cell; AST, aspartate aminotransferase; ALT, alanine aminotransferase; ALP, alkaline phosphatase; CRP, C-reactive protein; ICGR15, indocyanine green retention rate at 15 min; CEA, carcinoembryonic antigen; CA19-9, carbohydrate antigen 19-9.

There were significant differences between the two groups in the following parameters: BMI, WBC, neutrophil count, hemoglobin, platelet count, albumin, total bilirubin, ALP, CRP and CA19-9 levels. In brief, patients in the low mGNRI group had a lower BMI, lower hemoglobin and albumin levels, and higher WBC, neutrophil counts, ALP, and CRP levels than those in the high mGNRI group, consistent with poorer nutritional status and more pronounced inflammatory status.

Table 2 compares the surgical outcomes of the two groups. There were significant differences in surgical approach, tumor size, type of hepatectomy and lymph node metastasis.

**Table 2:** Perioperative characteristics of patients with intrahepatic cholangiocarcinoma (*N* = 99).

Variable	High mGNRI (*n* = 54)		Low mGNRI (*n* = 45)		*p*-value
**Surgical approach**					0.009
Open	33	(61%)	39	(87%)	
Laparoscopic	21	(39%)	6	(13%)	
**Type of hepatectomy**					0.006
Partial hepatectomy/segmentectomy	17	(32%)	3	(7%)	
Sectionectomy	11	(20%)	6	(13%)	
Bisectionectomy	21	(39%)	31	(69%)	
Trisectionectomy	5	(9%)	5	(11%)	
Extrahepatic bile duct resection, yes	6	(11%)	10	(22%)	0.222
Lymph node dissection, yes	30	(56%)	32	(71%)	0.166
Operative time, mins	372 (261–471)		396 (315–575)		0.115
Blood loss, mL	635 (303–1,219)		933 (521–1,232)		0.139
Blood transfusion, yes	20	(37%)	20	(44%)	0.588
**Tumor differentiation**					0.354
Well	12	(23%)	8	(18%)	
Moderate	34	(64%)	25	(57%)	
Poor	5	(9%)	10	(23%)	
Others	2	(4%)	1	(2%)	
Tumor size, cm	3.6 (2.5–5.0)		7.0 (3.7–9.0)		< 0.001
Lymph node metastasis, yes	7	(13%)	16	(36%)	0.016
**Number of tumors**					0.216
Solitary	49	(91%)	36	(80%)	
Multiple	5	(9%)	9	(20%)	
Serosal invasion, yes	17	(31%)	18	(40%)	0.502
Vascular and/or bile duct invasion, yes	46	(85%)	41	(91%)	0.555
Postoperative complications, yes	32	(59%)	27	(60%)	1.000
**AJCC staging**					0.063
I or II	34	(63%)	19	(42%)	
III or IV	20	(37%)	26	(58%)	
Adjuvant chemotherapy, yes	37	(69%)	26	(58%)	0.370
Deaths, yes	24	(44%)	33	(73%)	0.007
Recurrences, yes	28	(52%)	27	(60%)	0.542
OS time, months	33.7 (19.3–50.3)		24.3 (6.8–45.9)		0.034
RFS time, months	22.1 (10.2–39.4)		7.2 (3.6–22.2)		< 0.001

Data are presented as median (interquartile range) or *n* (%). mGNRI, modified geriatric nutritional risk index; AJCC, American Joint Committee on Cancer; OS, overall survival; RFS, recurrence-free survival.

Open hepatectomy, major hepatectomy, larger tumor size and lymph node metastasis were more frequent in the low mGNRI group. There were no significant differences between the two groups in short-term outcomes.

### 3.2 Univariate and multivariate analyses of mGNRI and recurrence-free survival and overall survival in intrahepatic cholangiocarcinoma

The median follow-up duration was 53.0 months (IQR, 35.2–93.2). During the follow-up period, a total of 67 RFS events and 57 OS events were observed among the 99 patients, with 32 patients censored for RFS and 42 patients censored for OS.

The Kaplan-Meier curves for RFS and OS in patients with high and low mGNRI groups are shown in Figure 2. The Kaplan–Meier estimated 1-, 3-, and 5-year OS rates for the entire cohort were 83.6, 49.0, and 37.0%, respectively. According to the mGNRI group, the corresponding OS rates were 72.3, 33.6, and 21.3% in the low-mGNRI group and 92.6, 62.5, and 52.6% in the high-mGNRI group. The Kaplan–Meier estimated 1-, 3-, and 5-year RFS rates for the entire cohort were 56.8, 33.7, and 30.4%, respectively. According to the mGNRI group, the corresponding RFS rates were 39.9, 23.3, and 18.1% in the low-mGNRI group and 70.4, 41.6, and 41.6% in the high-mGNRI group. The low mGNRI group had significantly worse RFS (HR: 2.09, 95% CI: 1.29–3.39, *p* = 0.003) and OS (HR: 2.25, 95% CI: 1.32–3.83, *p* = 0.003) compared with the high mGNRI group. As an additional analysis, the 3-year time-dependent ROC analysis identified an corresponding mGNRI cutoff of 91.05. Patients below this cutoff had worse OS (HR, 2.09; 95% CI, 1.21–3.61; *p* = 0.009). In 1,000 bootstrap resamples, 996 showed estimable results, with a median cutoff of 90.28 (95% bootstrap interval, 63.25–91.41) and a median HR of 2.59 (95% bootstrap interval, 1.57–6.08).

**Figure 2:**
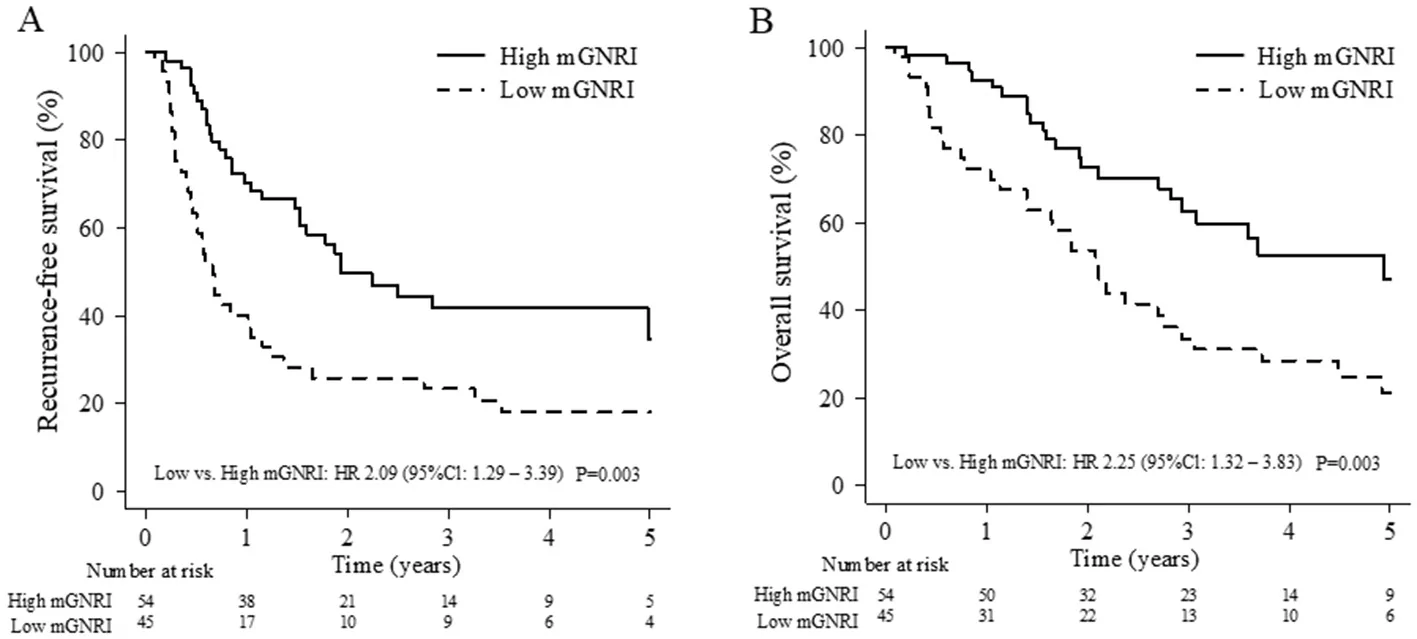
Kaplan–Meier curves for **(A)** recurrence-free survival (RFS) and **(B)** overall survival (OS) according to mGNRI group. The low mGNRI group showed significantly worse outcomes vs. the high mGNRI group for both RFS and OS.

The results of the univariate and multivariate analyses for RFS and OS are shown in Table 3. In the univariate analysis of RFS, a higher albumin level (≥ 3.9 g/dL) was associated with better prognosis (HR: 0.47, 95% CI: 0.29–0.76, *p* = 0.002). Accordingly, a low albumin level (< 3.9 g/dL), open hepatectomy, positive lymph node metastasis, and low mGNRI were considered poor prognostic factors. In the multivariate analysis, positive lymph node metastasis (HR: 2.35, 95% CI: 1.31–4.22, *p* = 0.004) remained an independent prognostic factor for RFS, whereas mGNRI did not retain statistical significance.

**Table 3:** Cox proportional hazards regression univariate and multivariate analysis of recurrence-free survival and overall survival in patients with intrahepatic cholangiocarcinoma (*N* = 99).

Variables	Recurrence-Free Survival						Overall Survival					
	Univariate analysis			Multivariate analysis			Univariate analysis			Multivariate analysis		
	HR	(95%CI)	*p*	HR	(95%CI)	*p*	HR	(95%CI)	*p*	HR	(95%CI)	*p*
Albumin (≥3.9 vs. < 3.9 g/dL)	0.47	(0.29–0.76)	0.002	0.63	(0.37–1.07)	0.087	0.43	(0.25–0.72)	0.002	0.54	(0.30–0.95)	0.031
Total bilirubin (≥0.6 vs. < 0.6 mg/dL)	0.97	(0.58–1.64)	0.911	1.10	(0.64–1.88)	0.733	1.05	(0.60–1.86)	0.863	1.40	(0.77–2.60)	0.274
Surgical approach (Laparoscopic vs. Open)	0.47	(0.26–0.85)	0.013	0.67	(0.34–1.31)	0.242	0.38	(0.19–0.78)	0.008	0.65	(0.29–1.43)	0.282
Type of hepatectomy (Major vs. Minor)	1.60	(0.95–2.68)	0.075	0.85	(0.45–1.58)	0.601	1.95	(1.08–3.52)	0.027	1.03	(0.52–2.05)	0.932
Lymph node metastasis (Positive vs. Negative)	3.27	(1.93–5.55)	< 0.001	2.35	(1.31–4.22)	0.004	2.79	(1.61–4.82)	< 0.001	1.93	(1.06–3.49)	0.031
mGNRI (Low vs. High)	2.09	(1.29–3.39)	0.003	1.63	(0.95–2.81)	0.078	2.25	(1.32–3.83)	0.003	1.86	(1.03–3.37)	0.041

mGNRI, modified geriatric nutritional risk index; HR, hazard ratio; CI, confidence interval.

In the univariate analysis OS, higher albumin level (≥3.9 g/dL), laparoscopic hepatectomy, minor hepatectomy, negative lymph node metastasis, and high mGNRI were associated with better survival. Accordingly, low albumin level, open hepatectomy, major hepatectomy, positive lymph node metastasis, and low mGNRI were considered poor prognostic factors. In the multivariate analysis, albumin level ≥ 3.9 g/dL was an independent protective factor (HR: 0.54, 95% CI: 0.30–0.95, *p* = 0.031), indicating that low albumin level was associated with worse OS. In addition, positive lymph node metastasis (HR: 1.93, 95% CI: 1.06–3.49, *p* = 0.031), and low mGNRI (HR: 1.86, 95% CI: 1.03–3.37, *p* = 0.041) were independent poor prognostic factors for OS.

The proportional hazards assumption was assessed using Schoenfeld residuals. The global test showed no significant evidence of non-proportionality for either OS (*p* = 0.82) or RFS (*p* = 0.088), although individual tests for mGNRI (*p* = 0.015) and surgical approach (*p* = 0.025) were significant in the RFS model (Supplementary Table S2).

To account for potential temporal confounding arising from changes in surgical and perioperative management over the long study period, a sensitivity analysis was performed adjusting for surgical approach, type of hepatectomy, lymph node metastasis, and calendar period of surgery. Low mGNRI remained significantly associated with worse OS (*p* = 0.046) and RFS (*p* = 0.041) after adjustment for these factors (Supplementary Table S3). However, in another sensitivity analysis using mGNRI as a continuous variable, mGNRI was not significantly associated with both OS and RFS after adjustment for the factors included in Table 3 (Supplementary Table S4).

### 3.3 Postoperative dietary intake status

Table 4 shows the average daily food intake over the four-day period from postoperative day 3 to day 6 for the two groups. All patients had complete oral energy intake measurements at all four time points; no missing repeated measurements were present. There were significant differences between the two groups in the following parameters: energy intake (kcal), fat intake (g), carbohydrate intake (g), and energy adequacy ratio (%). In addition, the oral energy adequacy ratio was compared between the two groups over the four days from postoperative day 3 to day 6. The oral energy adequacy ratio was significantly higher in the high mGNRI group throughout the entire period (Figure 3).

**Table 4:** Postoperative dietary intake assessment in patients with intrahepatic cholangiocarcinoma (*N* = 99).

Variable	High mGNRI (*n* = 54)	Low mGNRI (*n* = 45)	*p*-value
Energy intake, kcal	828 (500–1,038)	610 (339–809)	0.010
Protein intake, g	35 (23–45)	30 (15–39)	0.059
Fat intake, g	21 (12–28)	14 (9–24)	0.034
Carbohydrate intake, g	119 (69–156)	85 (46–118)	0.006
Protein adequacy ratio, %	56 (33–70)	46 (26–61)	0.051
Energy adequacy ratio, %	53 (30–65)	36 (22–50)	0.021

Data are presented as median (interquartile range). mGNRI, modified geriatric nutritional risk index.

**Figure 3:**
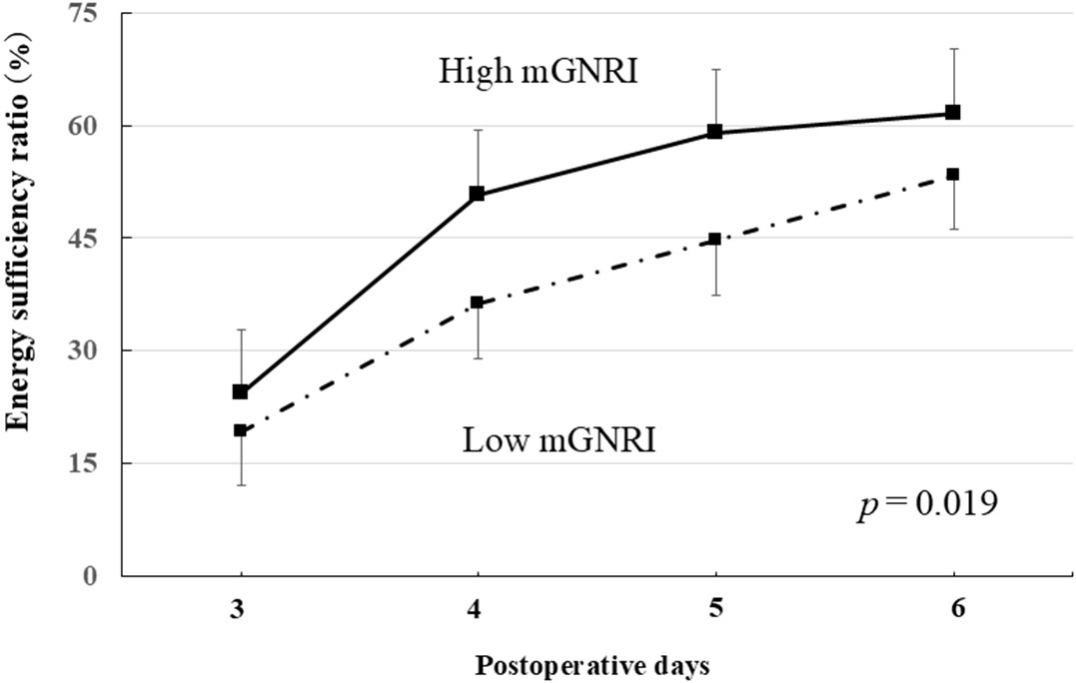
Postoperative oral energy adequacy ratio (%) from postoperative day 3 to day 6. The high mGNRI group maintained significantly higher energy adequacy ratios than the low mGNRI group throughout this period with main effect of mGNRI group (*p* = 0.019), postoperative time (*p* < 0.001) and mGNRI group × postoperative time interaction (*p* = 0.142).

## 4 Discussion

The results of this study showed the association between the preoperative mGNRI and long-term prognosis in patients with ICC after liver resection. When survival was compared between the high and low mGNRI groups, the low mGNRI group had a significantly worse prognosis for both RFS and OS. Although low mGNRI was not extracted as an independent prognostic factor for RFS, it was extracted as an independent poor prognostic factor in OS. The mGNRI has been reported to be associated with prognosis in esophageal cancer and pancreatic cancer (11, 12). To our knowledge, this is the first study to demonstrate that the mGNRI is associated with postoperative survival in patients with ICC undergoing liver resection and that it has independent prognostic value for OS.

Preoperative prognostication in the treatment of cancer is useful in reducing surgical risk and postoperative complications. In addition, the ability to predict prognosis by using non-invasive biomarkers is effective in reducing patient burden. Therefore, nutritional assessment in patients with cancer is important. Nutritional assessment methods can be broadly categorized into two types: Subjective Global Assessment (SGA) and Objective Data Assessment (ODA). SGA is a method of nutritional assessment based on a medical interview regarding body weight changes and dietary intake, while muscle mass, fat mass, and edema provide a detailed assessment of the body. ODA is an objective method of evaluation based on data from physical measurements, biochemical tests, and immunological tests, and it is important to evaluate these data comprehensively (20). Among ODA-based tools, simple composite indices that can be calculated from routine laboratory and anthropometric data are particularly attractive in daily practice.

The prognostic factors for cancer, the CAR, CALLY index, PNI, PLR, and NLR are indicators that combine biochemical and immunological data and are evaluated using ODA. On the other hand, the GNRI and mGNRI are indicators that are combined with biochemical tests and body weight and are also classified as ODA-based indices that reflect both nutritional reserve and systemic response to disease. The GNRI has been suggested to be associated with poor prognosis in a variety of diseases, including gastrointestinal cancers, such as esophageal, gastric, and pancreatic cancer, cardiovascular disease, and renal disease (13, 21–26).

However, serum albumin levels are affected by various factors, including a decline in liver function, infection, and inflammation. In particular, inflammatory diseases such as cancer promote the synthesis of CRP, which is known as an acute-phase reactant in inflammatory reactions, while at the same time suppressing the synthesis of serum albumin. For this reason, the European Society for Clinical Nutrition and Metabolism and the American Society for Parenteral and Enteral Nutrition guidelines state that serum albumin should not be used as a nutritional indicator (20, 27, 28). Therefore, the mGNRI, which combines CRP and body weight, is thought to be a suitable prognostic factor for patients with cancer. In ICC, CRP may be influenced by disease-related inflammatory conditions, including tumor progression and biliary tract inflammation. Therefore, mGNRI should be interpreted as an integrated marker reflecting both nutritional status and systemic inflammation rather than as a purely nutritional index. Its association with prognosis may also be influenced by the underlying disease severity.

Previous studies have reported that insufficient oral intake during the perioperative period of various cancer surgeries has a negative impact not only on nutritional status but also on postoperative survival (29–31). Okada et al. found that, at 4 weeks after surgery for esophageal cancer, the incidence of postoperative complications was significantly higher, and the survival rate was significantly lower in the group with poor oral intake (oral energy sufficiency rate of less than 25% compared with the required nutritional intake) than in the group with good oral intake (oral energy sufficiency rate of 25% or more). Furthermore, the postoperative oral intake rate was an independent prognostic factor for OS (29). These results indicate that perioperative oral intake is not merely a nutritional marker but is also closely linked to long-term oncologic outcomes.

In the present study, the postoperative oral energy intake and energy adequacy ratio (%) were significantly lower in the low mGNRI group than in the high mGNRI group (Figure 3). This suggests that patients with a low preoperative mGNRI are more likely to experience insufficient oral intake in the early postoperative period. The reason for the low oral intake may be a decrease in appetite due to inflammation, as the CRP level was already elevated prior to surgery (32, 33). In addition, the rate of open surgery was relatively high in the low mGNRI group. Compared to minimally invasive laparoscopic surgery, open surgery is associated with a higher degree of surgical stress and postoperative stress (34–37), and it was speculated that this may have led to a decrease in oral intake. These findings imply that a low mGNRI reflects a vulnerable host condition characterized by preexisting systemic inflammation, impaired appetite, and a greater likelihood of undergoing more invasive surgery, all of which may synergistically reduce postoperative oral intake and worsen outcomes. However, postoperative oral energy intake is influenced by multiple factors, including surgical approach and extent of hepatectomy, postoperative complications, medications, and patient- and care-related factors such as food preferences and the type and availability of food. Therefore, preoperative mGNRI may be associated with postoperative oral energy intake to some extent, although this association may also reflect the influence of these factors.

Recent studies have reported that the amount of oral intake increases after surgery when specialist staff are involved in rehabilitation interventions such as nutritional support, swallowing training, and meal assistance (38–40). Whether identifying patients with low preoperative mGNRI and providing targeted nutritional interventions in collaboration with dietitians can improve postoperative oral intake warrants further investigation. In patients with low mGNRI values, such nutritional interventions, if carried out before surgery, may help to improve the amount of oral intake after surgery. Therefore, our data support that mGNRI may serve as a tool to identify high-risk patients with ICC. Whether these patients benefit from targeted perioperative nutritional management requires prospective interventional evaluation.

In this cohort, because the extent of hepatectomy was closely associated with the preoperative mGNRI, the influence of surgical invasiveness could not be completely separated from host-related factors in this retrospective analysis.

This study has several limitations. First, there is a potential risk of selection bias due to the retrospective design and performance at a single facility. Second, the number of RFS events and the sample size were relatively small, which may have resulted in insufficient statistical power. Therefore, even if a true correlation exists between the mGNRI and RFS, it is possible that a statistically significant difference was not detected due to a β-error (41, 42). Third, the limited sample size may also have influenced the stability of multivariate models and restricted subgroup analyses, such as stratification by surgical approach or adjuvant therapy. In addition, because the extent of hepatectomy was closely associated with the preoperative mGNRI in this cohort, the effect of surgical invasiveness could not be fully disentangled from host-related factors in this retrospective study. Therefore, a multicenter prospective study is warranted to validate these findings. Moreover, the cutoff value of 88.2 was derived from the present cohort and should therefore be considered exploratory. In the additional 36-month time-dependent ROC analysis for OS, the corresponding cutoff value was 91.1, indicating some uncertainty in cutoff estimation. The association between mGNRI and overall survival was not statistically significant when mGNRI was analyzed as a continuous variable in the fully adjusted model; therefore, the prognostic significance of the ROC-derived cutoff of 88.2 should be interpreted with caution. In an additional 3-year time-dependent ROC analysis, bootstrap resampling showed a median cutoff close to the original estimate and a consistent association with OS. However, the wide bootstrap interval for the cutoff indicates uncertainty in the precise threshold, supporting the exploratory nature of this cutoff and the need for external validation. Therefore, external validation in an independent cohort is required before this cutoff can be applied in clinical practice. Future prospective studies should also investigate whether targeted nutritional and rehabilitation interventions in patients with low mGNRI values can improve postoperative oral intake and survival outcomes.

## 5 Conclusions

The mGNRI is a simple, non-invasive, and fully objective biomarker that can be calculated from routinely available clinical data. Our findings suggest that the mGNRI is a prognostic factor for ICC after hepatic resection. In particular, a low preoperative mGNRI value was independently associated with worse OS, and a higher preoperative mGNRI value was associated with better postoperative energy adequacy.

Furthermore, patients with a low preoperative mGNRI showed poorer survival and lower postoperative oral intake, highlighting the importance of perioperative nutritional assessment and the need for careful nutritional management in this population. The preoperative mGNRI may therefore serve as a tool to identify high-risk patients with ICC. However, whether postoperative nutritional intake contributes to or mediates the association between preoperative mGNRI and long-term survival remains unclear and warrants further prospective investigation.

## Data Availability

The raw data supporting the conclusions of this article are not publicly available to protect participant confidentiality and privacy. Requests to access the datasets should be directed to the corresponding author.
