# Supplementary material for: Modified geriatric nutritional risk index as a prognostic indicator for patients with intrahepatic cholangiocarcinoma undergoing liver resection
Source: Front Nutr. 2026 Oct 29;13:1965547. doi: 10.3389/fnut.2026.1965547 (PMC13758984; doi:10.3389/fnut.2026.1965547)
Supplement: Supplementary file 1 [file Table_1.docx]

Supplementary Table S1: Criteria for determination of activity and stress factors.

| **Activity level** | **Activity factor** |
| --- | --- |
| Bedridden with decreased consciousness | 1.0 |
| Bedridden | 1.1 |
| Bed rest | 1.2 |
| Activity outside the bed | 1.3 |
| **Clinical condition** | **Stress factor** |
| Chronic cerebrovascular disease | 0.9 |
| Mild postoperative stress | 1.1 |
| Mild infection | 1.2 |
| Moderate infection (sepsis), burn (40%) | 1.5 |
| Major postoperative stress (esophageal surgery) | 1.8 |

Supplementary Table S2*:* Assessment of the proportional hazards assumption for the Cox proportional hazards models using Schoenfeld residuals.

| **Covariate** | **OS p-value** | **RFS p-value** |
| --- | --- | --- |
| Albumin (≥3.9 vs. <3.9 g/dL) | 0.310 | 0.360 |
| Total Bilirubin (≥0.6 vs. <0.6 mg/dL) | 0.230 | 0.127 |
| Surgical approach (Laparoscopic vs. Open) | 0.790 | 0.025 |
| Type of Hepatectomy (Major vs. Minor) | 0.630 | 0.128 |
| Lympho node metastasis (Positive vs. Negative) | 0.210 | 0.218 |
| mGNRI (Low vs. High) | 0.590 | 0.015 |
| GLOBAL | 0.820 | 0.088 |

Supplementary Table S3. Sensitivity analysis using Cox proportional hazards regression for overall survival and recurrence-free survival adjusting for calendar period of surgery (N = 99)

| **Variables** | **Recurrence-Free Survival** | | | | | | **Overall Survival** | | | | | |
| --- | --- | --- | --- | --- | --- | --- | --- | --- | --- | --- | --- | --- |
|  | **Univariate Analysis** | | | **Multivariate Analysis** | | | **Univariate Analysis** | | | **Multivariate Analysis** | | |
|  | **HR** | **(95%CI)** | ***p*** | **HR** | **(95%CI)** | ***p*** | **HR** | **(95%CI)** | ***p*** | **HR** | **(95%CI)** | ***p*** |
| Surgical approach  (Laparoscopic vs. Open) | 0.47 | (0.26–0.85) | 0.013 | 0.66 | (0.34–1.29) | 0.221 | 0.38 | (0.19–0.78) | 0.008 | 0.60 | (0.27–1.32) | 0.200 |
| Type of hepatectomy  (H2–H3 vs. H0-H1) | 1.60 | (0.95–2.68) | 0.075 | 0.89 | (0.48–1.65) | 0.070 | 1.95 | (1.08–3.52) | 0.027 | 1.12 | (0.57–2.22) | 0.738 |
| Lymph node metastasis  (Positive vs. Negative) | 3.27 | (1.93–5.55) | ＜0.001 | 2.40 | (1.31–4.38) | 0.004 | 2.79 | (1.61–4.82) | ＜0.001 | 2.08 | (1.14–3.79) | 0.017 |
| Calendar period of surgery  (2016-2022 vs. 2008-2015) | 0.64 | (0.39–1.05) | 0.074 | 0.83 | (0.49–1.40) | 0.484 | 0.80 | (0.47–1.37) | 0.418 | 0.91 | (0.52–1.57) | 0.728 |
| mGNRI  (Low vs. High) | 2.09 | (1.29–3.39) | 0.003 | 1.75 | (1.02–3.01) | 0.041 | 2.25 | (1.32–3.83) | 0.003 | 1.81 | (1.01–3.25) | 0.046 |
| mGNRI, modified geriatric nutritional risk index; HR, hazard ratio; CI, confidence interval. | | | | | | | | | | | | |

Supplementary Table S4: Sensitivity analysis using Cox proportional hazards regression for recurrence-free survival and overall survival with albumin, total bilirubin and mGNRI as a continuous variable in patients with intrahepatic cholangiocarcinoma (N = 99)

| Variables | Recurrence-Free Survival | | | | | | Overall Survival | | | | | |
| --- | --- | --- | --- | --- | --- | --- | --- | --- | --- | --- | --- | --- |
|  | Univariate Analysis | | | Multivariate Analysis | | | Univariate Analysis | | | Multivariate Analysis | | |
|  | HR | (95%CI) | *p* | HR | (95%CI) | *p* | HR | (95%CI) | *p* | HR | (95%CI) | *p* |
| Albumin | 0.32 | (0.19–0.54) | <0.001 | 0.40 | (0.19–0.82) | 0.013 | 0.34 | (0.19–0.60) | <0.001 | 0.43 | (0.23–0.92) | 0.030 |
| Total bilirubin | 1.19 | (0.61–2.30) | 0.614 | 1.45 | (0.76–2.77) | 0.266 | 1.60 | (0.81–3.16) | 0.179 | 1.96 | (1.03–3.73) | 0.041 |
| Surgical approach  (Laparoscopic vs. Open) | 0.47 | (0.26–0.85) | 0.013 | 0.71 | (0.37–1.40) | 0.325 | 0.38 | (0.19–0.78) | 0.008 | 0.65 | (0.29–1.42) | 0.277 |
| Type of hepatectomy  (Major vs. Minor) | 1.60 | (0.95–2.68) | 0.075 | 0.89 | (0.49–1.61) | 0.703 | 1.95 | (1.08–3.52) | 0.027 | 1.17 | (0.61–2.25) | 0.629 |
| Lymph node metastasis  (Positive vs. Negative) | 3.27 | (1.93–5.55) | <0.001 | 2.31 | (1.27–4.20) | 0.006 | 2.79 | (1.61–4.82) | <0.001 | 1.84 | (1.01–3.36) | 0.046 |
| mGNRI | 0.97 | (0.96–0.99) | <0.001 | 0.99 | (0.98–1.01) | 0.504 | 0.98 | (0.96–0.99) | 0.005 | 1.00 | (0.98–1.02) | 0.669 |
| mGNRI, modified geriatric nutritional risk index; HR, hazard ratio; CI, confidence interval. | | | | | | | | | | | | |
